# Imaging Inflammation – From Whole Body Imaging to Cellular Resolution

**DOI:** 10.3389/fimmu.2021.692222

**Published:** 2021-06-24

**Authors:** Tuula Peñate Medina, Jan Philip Kolb, Gereon Hüttmann, Robert Huber, Oula Peñate Medina, Linh Ha, Patricia Ulloa, Naomi Larsen, Arianna Ferrari, Magdalena Rafecas, Mark Ellrichmann, Mariya S. Pravdivtseva, Mariia Anikeeva, Jana Humbert, Marcus Both, Jennifer E. Hundt, Jan-Bernd Hövener

**Affiliations:** ^1^ Section Biomedical Imaging, Molecular Imaging North Competence Center (MOIN CC), Department of Radiology and Neuroradiology, University Medical Center, Schleswig-Holstein Kiel University, Kiel, Germany; ^2^ Institute of Biomedical Optics, University of Lübeck, Lübeck, Germany; ^3^ Airway Research Center North (ARCN), Member of the German Center of Lung Research (DZL), Gießen, Germany; ^4^ Institute for Experimental Cancer Research (IET), University of Kiel, Kiel, Germany; ^5^ Department of Dermatology, Allergology and Venereology, University Hospital Schleswig-Holstein Lübeck (UKSH), Lübeck, Germany; ^6^ Department of Radiology and Neuroradiology, University Medical Centers Schleswig-Holstein, Campus Kiel, Kiel, Germany; ^7^ Institute of Medical Engineering (IMT), University of Lübeck, Lübeck, Germany; ^8^ Interdisciplinary Endoscopy, Medical Department1, University Hospital Schleswig-Holstein, Kiel, Germany; ^9^ Lübeck Institute for Experimental Dermatology (LIED), University of Lübeck, Lübeck, Germany

**Keywords:** MRI, PET, SPECT, optical imaging, Optical coherence tomography (OCT), precision medicine, Two-Photon microscopy (TPM), hyperpolarization

## Abstract

Imaging techniques have evolved impressively lately, allowing whole new concepts like multimodal imaging, personal medicine, theranostic therapies, and molecular imaging to increase general awareness of possiblities of imaging to medicine field. Here, we have collected the selected (3D) imaging modalities and evaluated the recent findings on preclinical and clinical inflammation imaging. The focus has been on the feasibility of imaging to aid in inflammation precision medicine, and the key challenges and opportunities of the imaging modalities are presented. Some examples of the current usage in clinics/close to clinics have been brought out as an example. This review evaluates the future prospects of the imaging technologies for clinical applications in precision medicine from the pre-clinical development point of view.

## Introduction

The frequency selective perception of electromagnetic waves is certainly one of the most astonishing achievements of evolution. The benefits of *seeing* were so striking that almost all species have picked up the concept in one way or another.

In medicine, visual inspection has always been the first line of assessing health and disease. As civilization advanced, so have the methods that help us *see*. Today, modern imaging methods allow us to visualize microbes, soft tissue, motion, specific antibodies, brain function, metabolism, and much more. An unprecedented plethora of imaging methods is available, not only to diagnose a patient, but to understand the mechanisms of life and disease.

New methods are being added to the quiver continuously, and hitherto inaccessible information becomes available. Different methods capture different aspects, and their combination adds up to a more complete picture of reality.

As treatment options explode, treatment control and choosing the right treatment for the patient becomes ever more important. Here, imaging is a key component to make *personalized medicine* come true: treating each patient effectively, efficiently, and individually.

Modern imaging methods, however, are just as complex as life and disease. Dedicated research communities have formed to face this challenge. In this review, we focus on the advances in imaging inflammation. It summarizes the results of the International Symposium (PMI 2020 Inflammation Medicine From Bench to Bedside) arranged by the German Excellence Cluster Precision Medicine in Chronic Inflammation (PMI) in Hamburg on 2020. For each methods, we provide a brief introduction into the technology and describe applications with respect to inflammation.

## MRI

Magnetic Resonance Imaging (MRI) is the gold standard when it comes to 3D, tomographic soft tissue, and functional imaging. Without ionizing radiation and only few contraindications it has become the method of choice for many diagnostic needs. Applications include imaging anatomy (1), flow (2), brain activity (3), microstructure (4), and, to some extent, metabolism (5) – all non-invasively and *in vivo*. To do so, MRI is taking advantage of the fact that nature has provided for small, magnetic sensors that are abundant in biological tissue – the magnetic moments of nuclear spins. The strongest magnetic moment of a stable atom is that of hydrogen ^1^H – the most abundant element in our body (approx. 10^25^ – more than all stars in the known universe). These magnetic moments can be excited by electromagnetic waves and emit a similar signal in return. These intrinsic sensors probe their surroundings and convey unique information that allows distinguishing tissues or molecules e.g. gray brain matter from the white one, or choline from creatine. From these data, the images (or spectra) are reconstructed.

Modern MRI systems can be programmed in many ways to yield images weighted by selected properties. Common examples include rather physical parameters such as T_1_, T_2_ or susceptibility weighting, physiological parameters such as perfusion, or structural parameters such as diffusion. While there is no dedicated “inflammation weighting”, some parameters were established as surrogate markers for inflammation; these will be discussed in the following.

While these images have shown great value for diagnostics, it should be kept in mind that they are not photographs but maps of abstract quantum mechanical or physical parameters. In the following, we review selected aspects of MRI with respect to imaging inflammation of vessels and the gut. In addition, we discuss the application of hyperpolarized MRI, which allows metabolic imaging in real-time.

### Imaging Perfusion in Inflammation With MRI

#### Background

The term perfusion refers to the transportation of oxygen and nutrients from the blood to tissues and organs by means of capillaries. In several brain diseases and pathologies, the blood supply is altered, which influences the perfusion of the affected areas. Therefore, the quantification of tissue perfusion provides valuable information to assess clinical diagnosis and medical treatment (6). Magnetic Resonance Imaging (MRI) can be used to measure perfusion levels without the use of ionizing radiation. Using this method, perfusion maps are calculated, providing a visual tool to support the clinical diagnosis of inflammatory brain diseases. By using MRI, the hemodynamics of perfusion can be described by means of various parameters, such as Cerebral Blood Flow (CBF) and Volume (CBV). Additionally, perfusion can also be characterized by the average time required for a particle (e.g., blood cell) to move through the vasculature (Mean Transit Time - MTT) and the particle velocity (7).

With MRI perfusion can be measured using exogenous or endogenous tracers. The most commonly used methods are Dynamic Susceptibility Contrast (DSC-MRI) (8), Dynamic Contrast-Enhanced (DCE-MRI) (9, 10)—both relying on the injection of a gadolinium-based external contrast-agent (CA)—and Arterial Spin Labelling (ASL) (11), which uses the water molecules in the blood as an endogenous tracer.

#### Applications

By tracking a CA bolus through the blood vessels, DSC-MRI reflects hemodynamic information as a hypointense signal in T_2_ or $T_{2}^{*}$ weighted images due to the increase in magnetic susceptibility of the CA in the blood (12). DSC-MRI is the standard for measuring perfusion in the human brain with MRI (12, 13), like in strokes and brain tumors. Additionally, this technique can provide information that helps differentiate malignant brain lesions such as metastases, lymphoma, and microvascular leakiness (14, 15).

DCE-MRI, sometimes called Permeability MR, is the standard approach for the measurement of perfusion outside the brain (16), e.g. in the liver (17) or prostatic (18). Here, the shortening of the relaxation time T_1_ by CA results in increased signal on, T_1_ weighted images, where the CA accumulates. The time course of the MR signal, reflects the response of the target tissue to the CA’s arrival. Providing quantitative information on the integrity of the Blood-Brain Barrier (BBB), tumor growth factors, and response to treatment.

The BBB permeability is the main neuroinflammatory phenomenon that can be assessed with DCE-MRI (19), for example, while monitoring the active phases of multiple sclerosis. In principle, the integrity of the BBB does not define an inflammation process “per se”, but most of the neuroinflammatory activity affects the integrity of the BBB (20).

In contrast, ASL uses magnetically labeled water in the blood as an endogenous CA. There are several ASL techniques—which mainly differ on the characteristics of the labeling method—with pseudo-continuous ASL (pCASL) (21) being the method of choice in the clinical routine (22). It is an entirely non-invasive technique, able to provide absolute values of blood perfusion in tissue. By using this technique, it is possible to obtain perfusion territory maps that can provide invaluable information for the treating, planning and monitoring of cerebrovascular diseases, tumour blood supply, and vessel malformation (23). Recent advances in ASL aim to identify the specific territory that is supplied by a specific artery. This territorial-ASL, also called selective-ASL, allows to determinate and visually pinpoint not only perfusion territories but also flow, providing patient-specific information for the diagnosis of cerebrovascular disease (23).

### Imaging Inflamed Vessel Walls

#### Background

For the workup of intracranial arteriopathies, conventional angiographic methods, including Digital Subtraction Angiography (DSA), Computed Tomography (CT), and magnetic resonance imaging (MRI) are routinely employed. Still, these methods can only depict the lumen and evaluation of disease status and progression depends on the extent of change in luminal diameter. For the differential diagnosis of arteriopathies, visualization and analysis of the artery walls can provide valuable information. While direct measurement of wall thickness is not possible in clinical MRI scanners due to the limited spatial resolution, visualization of diseased thickened or contrast-enhancing vessel segments is feasible with MR Vessel Wall Imaging (VWI). To depict the arterial wall, a high contrast between the vessel lumen and the wall is needed. The signal from flowing blood can be suppressed by special MR sequences, so-called Black-Blood MR imaging (BB MRI), increasing the contrast between the vessel wall and lumen. A frequently used sequence for BB MRI is a pre-and postcontrast 3D T1-weighted fast spin-echo sequence (3D T1 FSE), which effectively suppresses the signal from flowing blood and providing full brain coverage within an adequate examination time (24, 25). The signal from flowing blood is suppressed primarily by intra-voxel signal dephasing due to the velocity distribution within the imaging voxel and the outflow of the blood from the imaging slice during examination (26, 27). To improve blood signal suppression further, the sequence can be complemented by additional flow suppression modules (28, 29).

To reduce the artificial thickening of the vessel wall due to partial volume effects, a sufficiently high submillimeter resolution is required. However, the high spatial resolution comes at the cost of a low Signal to Noise Ratio (SNR) and a longer examination time, which might lead to motion artefacts. Thus, commonly used isotropic voxel sizes range between 0.5 – 0.8 mm^3^. MR imaging at higher magnetic fields (7T or more) can improve the SNR (30). The development of new acceleration techniques can reduce the examination time (31) while maintaining or increasing the spatial resolution.

#### Application

**Figure 1:**
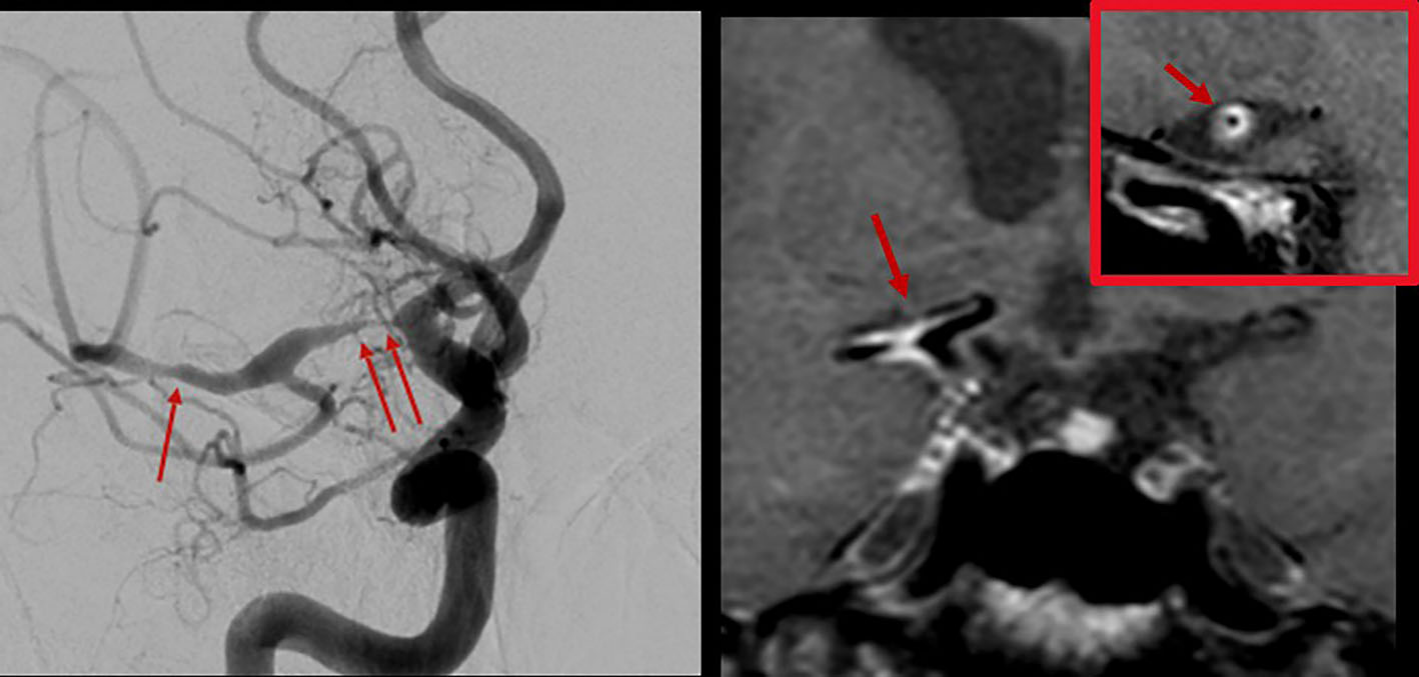
Digital subtraction angiography of the brain with injection in the right internal carotid artery in a patient with varicella-zoster vasculitis (left). Multiple stenoses in the M1 segment of the right middle cerebral artery were found (arrows). 3T MR vessel wall imaging (right) shows strong contrast enhancement of the corresponding segments (arrows). The inset shows a transverse section through the proximal M1 segment with circumferential wall enhancement pattern.

*Vasculitis:* Central Nervous System (CNS) vasculitis is categorized as either idiopathic Primary Angiitis of the CNS (PACNS), as CNS manifestation of systemic rheumatologic diseases or associated with infection. The diagnosis of PACNS is challenging since valid biomarkers are not available. PACNS can present with a wide range of nonspecific symptoms like headache, stroke/transient ischemic attack, cognitive dysfunction, and seizures. Treatment options include glucocorticoids, immunosuppressive agents as cyclophosphamide, and the anti-CD20 monoclonal antibody Rituximab. The diagnosis is mainly based on cerebrospinal fluid analysis, typical findings in MRI and DSA, and biopsy. Imaging plays an important role in the exclusion of differential diagnoses. Digital subtraction angiography can reveal typical findings (**Figure 1**, left) but is reported to have low sensitivity and specificity (32). In the recent past, MR VWI has emerged as an important supplementary tool not only for the detection of parenchymal changes but for the improved visualization of the vessel wall and vessel pathology in the differential diagnosis of CNS vasculitides. In acute vasculitis, the arterial wall appears circumferentially thickened and strongly and homogeneously contrast-enhancing (**Figure 1**, right). The pattern of distribution in the cerebral vasculature is typically multifocal and segmental. In patients presenting with stroke, VWI findings can aid in distinguishing vasculitis from other etiologies, including intracranial atherosclerosis and reversible cerebral vasoconstriction syndrome (33–38), and in monitoring the therapy response. Moreover, MR VWI can assist in identifying a target lesion if a biopsy is indicated.

**Figure 2:**
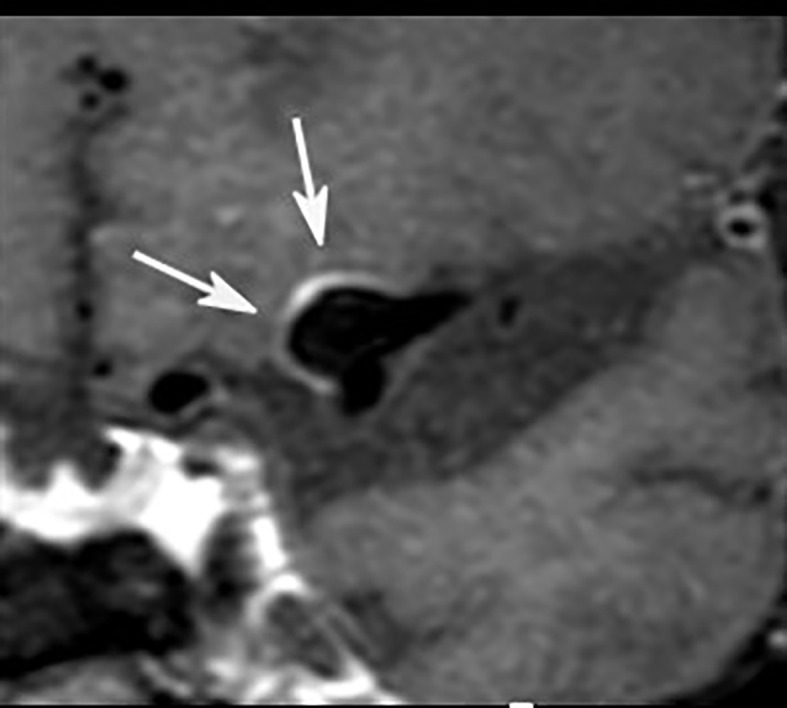
Postcontrast 3T MR vessel wall imaging of a 5 mm aneurysm at the middle cerebral artery bifurcation. Note the strong wall enhancement (arrows) as a possible marker for visualization of wall inflammation.

*Intracranial Aneurysms*: Aneurysms of the intracranial arteries were reported to have a prevalence of up to 3%. They are often incidental findings on neuroimaging and generally harbor a low risk of rupture (39, 40). In the case of a subarachnoid hemorrhage following rupture of an intradural aneurysm, a devastating outcome with persistent severe neurological deficits or even death is frequent (41). Therefore, risk stratification of patients diagnosed with an unruptured intradural aneurysm is crucial, but optimal management remains controversial. Recently, wall enhancement in intracranial saccular aneurysms on MR vessel wall imaging has been associated with a higher risk for rupture (42–46) (**Figure 2**). Experimental studies indicated that flow triggered inflammation in the vessel wall (specifically, macrophage invasion in the vessel wall) may cause formation and growth of intracranial aneurysms (47–52). Moreover, recently published results found an association of inflammatory processes in the aneurysm wall with contrast enhancement on MR VWI (45, 53–56). Therefore, wall enhancements may serve as a biomarker for inflammatory processes associated with wall destabilization and a higher risk for rupture of intracranial aneurysms and could aid in the risk stratification of patients with an incidental aneurysm.

### Imaging of Small and Large Bowel in Patients With Inflammatory Bowel Disease (IBD)

**Figure 3:**
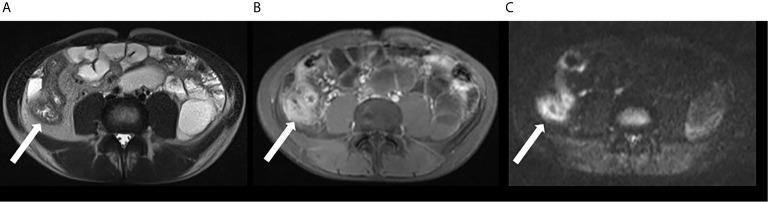
MRI in patient with active CD involving the ileum: there is bowel wall thickening in T2w sequence **(A)** and increased contrast media uptake in T1w, fat suppressed imaging **(B)**. Inflammation is also revealed by hyperintensity in DWI **(C)**.

Inflammatory Bowel Diseases (IBD), including Ulcerative Colitis (UC) and Crohn’s Disease (CD), are chronic inflammatory disorders characterized by sequences of flares with active symptomatic disease and periods of remission. While UC is typically restricted to inflammation of the mucosa and the submucosa of the large bowel, CD is a transmural process with manifestation in the gastrointestinal tract from the mouth to anus, predominantly at the terminal ileum, inducing stenoses and fistulas. IBD are disabling, life-long disorders associated with an increased risk of colorectal cancer. Typical medications include steroids, 5-aminosalicyclic acid products, immunomodulators, and biologicals like Tumor Necrosis Factor (TNF) inhibitors. Complicated disease courses require a surgical procedure (57, 58). Besides clinical and serological assessment, endoscopy and video capsule endoscopy, cross-sectional imaging, including MRI, CT, and ultrasound (US), is crucial in setting IBD as first-line techniques in diagnosis, staging, and follow-up under medical therapy (59). CT enterography and MR enterography provide comparable diagnostic performances in patients with CD (60). Nevertheless, recent studies advise preferring MR enterography because of the absence of ionizing radiation, a very high soft-tissue contrast, and a lower incidence of adverse events (61). Although MRI and US are regarded as complementary methods in CD (59), most studies revealed superior accuracy of MRI for detecting the presence, extent, and activity of small bowel CD disease compared to US (62). In general, MRI protocol comprises fastening and application of hyperosmolar oral contrast agents like mannitol prior to the examination. Typical MRI sequences are axial and coronal Fast Spin-Echo (FSE) T2W sequences with and without fat saturation, axial and coronal Steady-State Free Precession Gradient Echo (SSFP GE) sequences without fat saturation, and non-enhanced coronal T1W sequence with fat saturation followed by contrast-enhanced coronal and axial T1W sequences with fat saturation. Free-breathing Diffusion-Weighted Imaging (DWI) sequences (**Figure 3**) are optional (63). MRI findings of active CD include segmental wall-thickening and hyper-enhancement after gadolinium-based contrast media, edema, strictures, ulcerations, restricted diffusion, sacculations, enlarged local lymph nodes and hypervascular appearance of the mesentery (comb sign). Several MR scoring systems have been developed to measure disease activity, e.g., the Magnetic Resonance Index of Activity (MaRIA score) (64). Due to side effects of gadolinium-based contrast media – cerebral deposition and nephrogenic systemic fibrosis – native techniques have gained increasing interest in MRI (65). In recent studies, DWI was found to be mostly equal to contrast-enhanced MRI in die assessment of IBD (66, 67). There are also promising data regarding sophisticated DWI methods in the assessment of disease activity in IBD, like Diffusion Kurtosis Imaging (DKI), which reflects the heterogeneous water diffusion behaviour more accurately compared to standard DWI (68). Together with innovative tools for quantifying bowel motility (69) in patients with CD, these techniques could offer the opportunity to establish valid non-contrast-enhanced MRI protocols for patients with IBD.

### Imaging Inflammation With Hyperpolarized MRI

#### Background

All of the MRI methods mentioned above offer unique insights into the biology and the functions of the human body. Still, many MRI applications fall short of their potential because scan times are too long or the signal to noise ratio (SNR) is too low. For example, MR is unique in being able to measure the biochemical composition of tissue non invasively and *in vivo*. This technique, MR-spectroscopy, has found important applications, but suffers from low chemical and spatial sensitivity, while the scan times are long (70–72).

At the same time, the early diagnosis of diseases before macroscopic pathologies occur is direly needed (e.g. tumors, aneurysm or chronic inflammation).

As a consequence, much research is focused on improving MRI. Most of these methods offer fractional improvements, e.g. a SNR gain of 30% by acceleration techniques. Other provide a few-fold enhancement, e.g. by increasing the magnetic field from 1.5 T to 3 T and 7 T. MRI with hyperpolarized contrast agents, however, has demonstrated to boost the signal by several orders of magnitude – e.g. 10.000 or 100.000 fold of the selected molecules. Like MRI, hyperpolarization is an inherently quantum mechanical effect. As described above, MRI is based on the magnetic moment of atomic nuclei, which is induced by nuclear spins. In some aspects, this magnetic moment behaves like the needle in a hiking compass. Like a compass, the nuclear spins align in an outer magnetic field; unlike a hiking compass, however, the spins don’t all align in the same direction. Instead, the spins are distributed in parallel or antiparallel to the magnetic field following the Botzmann distribution. As spins pointing up and down cancel, only the population difference effectively contributes to MRI signal. The fraction of all spins contributing to the signals is called polarization (P):

$$P=(N\beta -N\alpha )/(N\beta +N\alpha )=\tanh(\gamma =B_{0}/2k_{B}T)\approx \gamma =B_{0}/k_{B}T$$

Where N is the occupation number of the upper and the lower energy levels α and β, ħ is the Planck constant, γ is the gyromagnetic ratio, B_0_ is the magnetic field applied, k_b_ is the Boltzmann constant, and T is the thermodynamic temperature.

For all practical matters *in vivo*, the polarization is very, very small. In the magnetic field of the earth, ≈ 50 μT, the polarization is only a few parts in a billion – only few ppb contribute effectively to the MR signal. In a magnetic field of 1.5 T, the fraction is increased to a few in a million. In other words: 99.999% of all spins in a sample (or body) are invisible in routine MRI, leaving room for a dramatic enhancement of the MR signal and new diagnostic applications.

Several methods have been developed to increase the polarization of a solid, liquid, or gas (73–75). Usually, these methods use some spin order that is readily available in nature to increase the polarization of the target substance. For biomedical applications, hyperpolarized metabolites and gases are particularly interesting.

#### Applications

Biomedical MRI of hyperpolarized metabolites in solution was introduced in the early 2000s. Here a hyperpolarized CA is injected *in vivo* and several metabolites are measured (e.g. pyruvate to lactate, alanine, bicarbonate). Since then, impressive works have been published, including the first applications to humans, where real-time metabolism was detected in the brain and heart (76, 77). In the prostate, cancerous metabolism was detected before visible lesions occurred - an important step for early diagnosis and personalized medicine (76).

**Figure 4:**
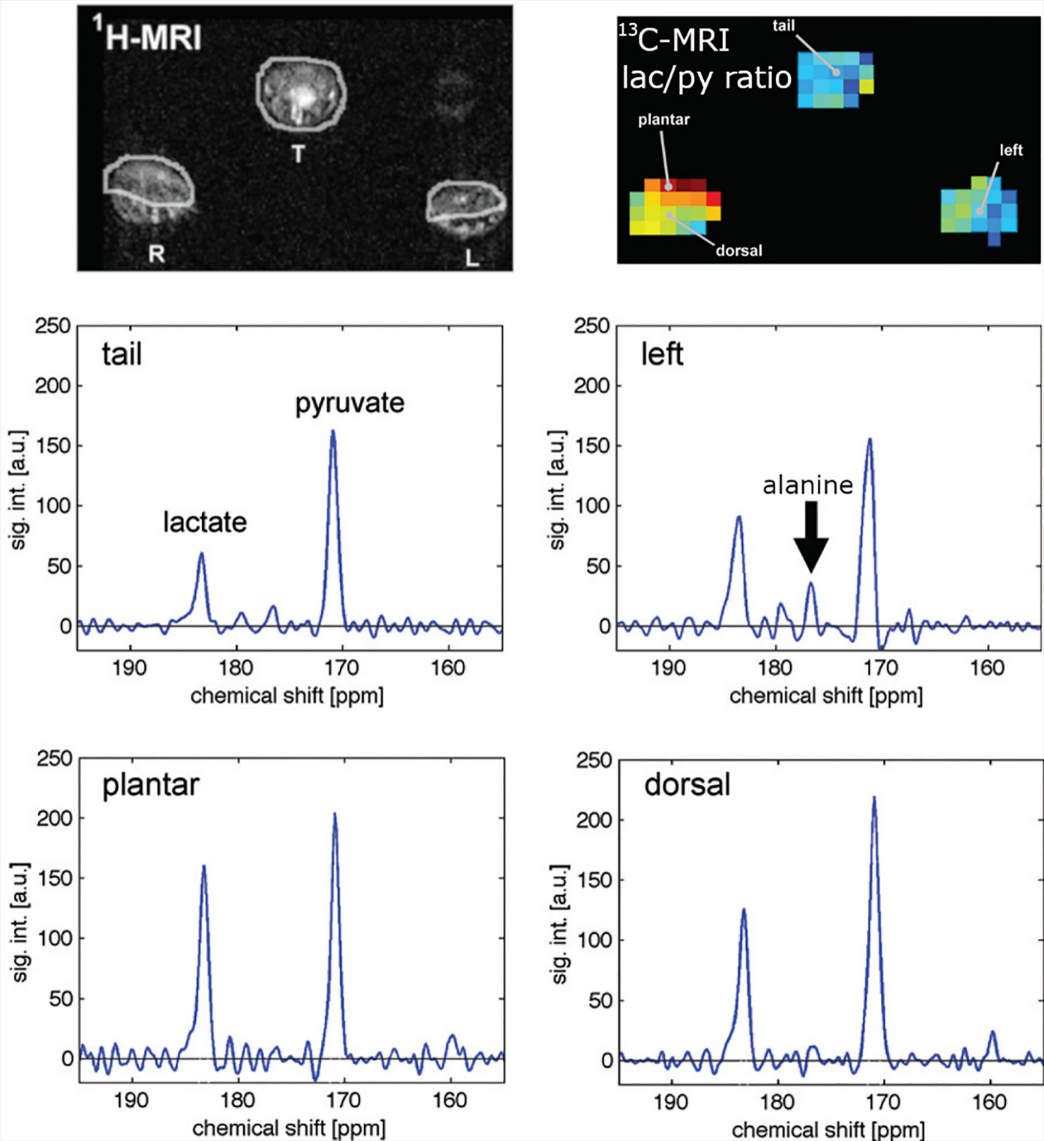
Measuring the metabolism of an arthritis model with hyperpolarized hyperpolarized MRI: anatomical 1H MRI (top left), quantitative metabolic map of lactate-to-pyruvate ratio (top right) and corresponding 13C spectra. Arthritis was induced in the right paw of the rats while the left served as a control. Hyperpolarized pyruvate was injected and ^13^C metabolic imaging performed. The inflamed paw exhibited a 65% increase in lactate signal and no alanine signal indicating abnormal metabolism. Figure modified from [MacKenzie et al. (79)].

As for inflammation (78), it was found that the inflamed paw of an arthritis model (79) showed a higher pyruvate to lactate ratio than the control paws (**Figure 4**). The higher amount of lactate correlated to inflammation, as was validated by clinical and histological analysis. In another study the same CA (hyperpolarized pyruvate) was used to assess hepatocytes necrosis in a CCl_4_ rat model. The conclusions was that ^13^C metabolic imaging with hyperpolarised [1-^13^C] pyruvate is sensitive to inflammation (80). Lewis and co-workers showed that hyperpolarized [1-^13^C]pyruvate can be used to evaluate the local cardiac inflammatory response due to Myocardial Infarction (MI) (81) with a broad potential across cardiovascular diseases.

Eto and co-workers (82) followed a different approach and used radicals *in vivo* for redox imaging in skeletal muscle disorders associated with inflammation.

**Figure 5:**
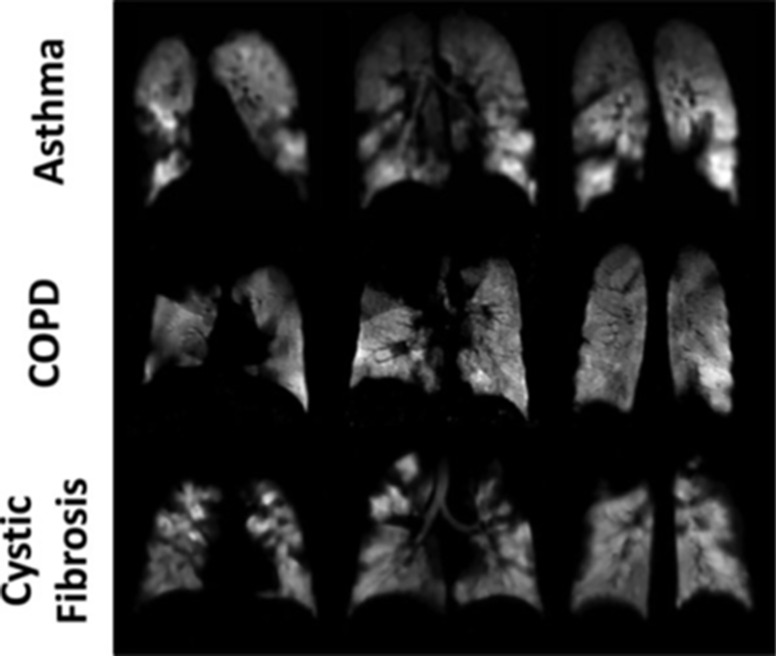
Ventilation imaging of the diseased human lung using ^129^Xe-MRI. Coronal ventilation images were acquired in subjects with asthma (upper row), COPD (middle row), or cystic fibrosis (lower row). Numerous ventilation defects can be seen in each of the images secondary to airflow obstruction caused by the underlying diseases. Figure taken from Mugler, J. P. et al., Journal of Magnetic Resonance Imaging (85).

MRI with hyperpolarized gases, Xenon-129 (83) and Helium-3 (84), provides unique diagnostic information on the human lung (85, 86). Imaging the gas distribution provides ventilation maps in 3D with high resolution (**Figure 5**), measuring the diffusion allows to assessing the lung microstructure, e.g. the alveolar condition (87). The gas exchange and function of the lung can be measured by using spectroscopic MRI, where Xenon in the airspaces can be distinguished fron Xenon dissolved in blood plasm and bound to red blood cells (88). These techniques were used to access chronic obstructive lung disease (COPD) (89), asthma (90), idiopathic pulmonary fibrosis (91) and a local inflammation (92). For example, ventilation deficits can be readily imaged with ^129^Xe-MRI. Likewise, **Figure 5** depicts clearly the lung degradation with different pulmonary diseases obtained by ventilation ^129^Xenon MR-imaging.

## Emission Tomography: PET and SPECT

### Background

Positron Emission Tomography (PET) and Single Emission Computer Tomography (SPECT) are well-established imaging techniques in both clinical routine and pre-clinical research for a large variety of applications. PET and SPECT rely on the administration of specific radiotracers and subsequent detection of high-energy photons. Both modalities stand out for a superb sensitivity, which translates into the detection of radioisotope concentrations in the nano to picomolar range. In the case of SPECT, the selected molecules or particles are labeled with gamma-emitting radioisotopes, whereas for PET positron-emitting radioisotopes are required. The emitted positrons are not directly detected by the scanner, but the pairs of high-energy photons that arise from the interaction between positrons and their counterparts, the electrons from the tissue. Thanks to this feature, PET offers a higher efficiency than SPECT, as the latter requires collimators to select only those photons from a certain direction. In any case, the detected photons indirectly reveal the location of the radiotracers. To extract this information, tomographic image reconstruction is required,

The radioisotopes Technetium-99m (^99m^Tc) and Fluorine-18 (^18^F) have remained for decades as workhorses for PET and SPECT, respectively. The latter is mainly used to label Flurodeoxyglucose (FDG); the resulting tracer ^18^F – FDG is a commercially available glucose surrogate, and as such, it has been successfully employed to track glucose metabolism within a large variety of diseases. Additionally, a large variety of radioisotopes can be used for labeling relevant substances, from simple molecules such as water, to antibodies, drugs and even bacteria. As the radiotracers are designed to target selected biochemical processes, their distribution in time and space unveil the underlying metabolism and biokinetics. Theranostics approaches go one step beyond, so that the radiolabelled compounds, designed e.g. to irradiate malignant cells, can be also localized by means of PET or SPECT.

At present, stand-alone PET scanners have become a rarity, and bi-modal PET/CT and PET/MRI systems are used instead, not only in the clinics but also for small-animal imaging. Also, SPECT/CT scanners are commercially available; SPECT-MR still remains only restricted to rodents, although some developments aimed to bring SPECT/MR into the clinics have been reported (93). All these synergistic approaches offer both functional and anatomical information. Moreover, the information provided by additional modality helps enhance the quality of the PET or SPECT images. This, in turn, leads to improved lesion detection and, in the case of PET, more accurate quantification. In the context of imaging inflammation and infection, simultaneous PET/MRI has proved to be advantageous compared to independent scans of the two modalities (94). One concern for CT as additional modality is the increased total radiation exposure. This is obviously not the case for PET/MRI, as MRI does not require ionising radiation. In any case, latest advances in instrumentation and software have contributed to significantly reduce the delivered effective doses without jeopardizing image quality.

Whereas visual interpretation of PET and SPECT images usually suffices for routine diagnostics, PET (and to a lesser extend also SPECT) can also provide quantitative information. Quantitative PET mainly refers to extracting from the reconstructed images the absolute amount of radiotracer accumulated in a specific region of interest within a certain time frame. This information can be expressed in terms of e.g. kBq/ml, or as a standardized uptake value and can be particularly useful to assess the response to therapy. Furthermore, the rate of tracer transportation and exchange (tracer pharmacokinetics) can be estimated from dynamic PET data in combination with kinetic modeling analysis.

Producing a PET image from the measured data is a complex process. Thanks to the increasingly growing computing power of desktop PCs and GPUs, the time required for image reconstruction has been strongly reduced. Advanced algorithms have become part of the manufacturers’ software so that images are generated shortly after the scan is completed or even on-the-fly. Still, there is room for further improvements at the software level, which should go hand in hand with the corresponding advances in instrumentation to fully exploit the potential of novel components and designs (95). The recent development of Total-Body (TB) PET scanners (96, 97) is expected to boost molecular imaging and personalized medicine. Such systems allow the entire patient body to be imaged in a single scan, making a further dose reduction and faster imaging possible (e.g., 1-min scans). In particular, TB-PET opens the door to ultrahigh-resolution dynamic imaging with 100-ms short frames to capture the fast initial distribution of the radiotracer. In the last decades, organ-specific imaging devices as well as systems for intraoperative use have been developed although very few imaging concepts have reached commercial maturity. In contrast, dedicated rodent scanners, developed to provide high sensitivity and high spatial resolution, are long commercially available to support preclinical research. Current small-animal PET scanners are characterized by a spatial resolution of about 1 mm, whereas a better resolution (but worse sensitivity) can be achieved by pre-clinical SPECT systems. The progressive consolidation of zebrafish as a model organism for pre-clinical research, also to investigate inflammation (98, 99), is demanding the availability of specific PET systems and protocols (100). In this vein, some of us have started developing a dedicating system and imaging setup to allow for zebrafish PET imaging (101).

### Applications

Diagnostic PET and SPECT are routinely employed in oncology, cardiology and neurosciences. Their suitability to image inflammation has been long recognized (102–106), also specifically for inflammatory bowel disease (107–115), including preclinical research on murine models (116, 117).

The specificity of PET and SPECT relies on the choice of the radiolabelled compound. Several radioactive tracers have been used for the detection of the immune system and inflammation. The gold standard of lymph node detection in surgical settings is based on the Tc-99 sulfo-colloid nanoparticle. These technetium levels are measured *via* gammascintillation counter from surgical samples or *in situ* by using gamma camera or SPECT. The sulfo-colloid meshwork role is needed to slow down the radio-ligand diffusion and ensure that the elimination of the complex will be done through the lymphatic drainage *via* lymph nodes. In this method, the technetium sulfo-colloid is injected several hours in advance of the surgical operation, and the imaging is done prior to the procedure. However, there is often no additional help to the surgeon in an intraoperative setting except the possibility to scan individual lymph nodes at the site with a radioactive detection device. An injection of optical tracer, Cardiogreen (ICG), has been used to bring this component to the surgery. The ICG injection could help to detect sentinel lymph nodes in intraoperative settings, but it can be used reliably only after skin removal and up to 1-1.5 cm deep into the tissue (118, 119). Alternatively, mini gamma cameras or freehand SPECT systems could be used. These devices have been introduced for intraoperative applications, although their use is not widespread (120).

^18^F-FDG has been used successfully for the detection of highly active inflammation. The use of FDG is based on the fact that FDG resembles glucose enough that it is internalized by the cells that are in need of glucose. FDG cannot be further metabolized, like glucose, leading to FDG accumulation. The PET tracer ^18^F-FDG thus allows cell imaging and cell labeling, while several different cells take it up efficiently. Macrophage labeling has been used to track the status of inflammation in arthritic patients. The macrophages are first extracted from the blood, labeled with a radioactive tracer, and reinjected into the bloodstream to follow the accumulation to the organ of interest. Several inflammatory disorders include sarcoidosis, atherosclerosis, vasculitis, IBD, rheumatoid arthritis (RA), and degenerative joint disease are imaged using immune cells. Gallium-67 (^67^Ga) citrate, ^99m^Tc- or ^89^Zr-labelled leukocytes, indium-111 (^111^In), as well as ^18^F-FDG represent the most widely used radiopharmaceutical agents (115, 120–122). In addition to cells, bacteria have been targeted and imaged by using radiolabeled antibiotics (123). However, other preparations, like labeled murine monoclonal antigranulocyte antibodies and labeled human polyclonal nonspecific immunoglobulin G, chemotactic peptides, interleukins, chemokines, and liposomes, have been used to image inflammation (124–127). Chelates that can be coupled to different proteins, lipids, and sugars are widely used in the development of new tracers. At another level, the combination of PET with radiolabeled therapeutic agents, such as liposomal glucocorticoids, is helping to push forward drug development in the treatment of inflammatory diseases (128). It is thus to be expected that current advances in radiochemistry and radiopharmacy, together with improved imaging technology, will further contribute to consolidate PET and SPECT as indispensable tools for precision medicine.

## Optical Imaging

Optics covers some of the oldest and most important forms of medical diagnosis and research. By simply looking at a patient, the shape and color perceived with the naked eye can already provide valuable diagnostic information. The strength of optics in biomedicine is its potential for very high spatial resolution and specific contrast. Optics is capable of visualizing sub-cellular structures and stood at the beginning modern medicine. Today, optical microscopes can resolve even structures only a couple of 10 nanometers in size and using fluorescence techniques, they can provide molecular functional contrast. Since they do not use ionizing radiation or particle beams, optical microscopes exhibit very good non-destructive and even *in vivo* capabilities in contrast to other high-resolution techniques like for example electron microscopes or micro CT.

Thus, by now optical imaging and sensing is of paramount importance in clinical and medical research laboratories in form of benchtop devices. These range from standard types of reflection, transmission and fluorescence microscopes to more advanced confocal, two photon and Stimulated Emission Depletion (STED) or Photo‐Activated Localization Microscopy (PALM) super resolution microscopes. But also devices like flow cytometers and cell sorters and almost all DNA sequencers use optical methods – mainly fluorescence – for sensing. Right now, the digital revolution, which enables fully electronic processing of images and photos in consumer products, is starting to have a massive impact on medical imaging (digital microscopes, camera in a pill etc.). Supported by the new possibilities offered by modern data processing units, the rise of optics in medicine will continue.

Considering *in vivo* imaging applications in a clinical setting, the eye and the skin are ideal target organs since they are very easily accessible by optical technologies. With respect to other target organs, besides the numerous biophotonic laboratory tools mentioned above to sense extracted samples or cells outside the body, the main problem of optical *in vivo* imaging for diagnosis in patients is the poor penetration of light into highly scattering tissue. Still, in many cases, it is possible to use ***endoscopes in order to deliver light to deep inside the human body***. Hence, almost all epithelial structures at “barrier interfaces” are accessible by current endoscopes. Today’s endoscope technology in clinical routine almost exclusively performs simple reflection imaging, which means, simple color images of the sample are created. However, there are more advanced optical imaging techniques as mentioned above, which could provide an additional wealth of information for an earlier and more precise diagnosis of disease.

Generally speaking, it were always advances in technology which triggered a paradigm shift in the medical application of optics and opened new realms of application to use optics as tool for early diagnosis of disease, supporting treatment decisions and monitoring patient response. Recently especially with the advent of full digital imaging processing chains optical imaging is not only used to generate image but also to quantify disease stages by deriving robust parameters like layer thicknesses, vascular branching densities or tissue elasticity. This allows an observer independent quantification of health or disease status for more consistent and precise treatment decisions.

In the following, several examples spearheading the introduction of advanced optics to inflammation are described.

### Fluorescent and Molecular Imaging

#### Background

Clear advantages for optical imaging are the fact that optical imaging devices and patient imaging are, in general, cheaper than radioactive and MRI imaging. Optical imaging is sensitive, and the theoretical resolution is excellent, allowing accurate molecular imaging. Optical imaging can also be performed more often in one person in contrast to CT or radioactive imaging, which are limited due to maximum radiation dosages. Some methods like ICG based rheumatoid arthritis imaging are in sporadic use in clinics (129). The main reason that hinders the usefulness of optical imaging in daily practice is the limited penetration depth. The optical signal can travel only a couple of centimeters at best when using Near-Infrared (NIR) wavelengths and NIR probes. This distance can theoretically be extended up to 10 cm when photoacoustic detection is used (130). Photoacoustic Imaging (PAI) combines light and ultrasound into an absorption-based non-invasive imaging technique. In PAI the ultrasound signal emerging from the thermoelastic expansion caused by optical absorption within biological tissues or the contrast agent is measured. These spatial limitations of the penetration depth are less relevant in small animal imaging, where full 3D tomography can be performed due to the small size of the animals of interest. Clinically approved fluorophores can be sensitive to their environments and give different readings based on their surroundings (131). At the moment, one brand of 3-D fluorescence optical tomography is available for a small animal. These include mice, hamsters, rats, and small rabbits. For PAI, pre-clinical instruments for small animals can be coupled with co-registered ultrasound imaging, yielding a 2D or a 3D-tomograpic image with anatomical and molecular information (132). Complicated immune reactions can be studied in disease models like Dextran Sodium Sulfate (DSS) inflicted acute and chronic IBD mouse models (133). Several ready-made NIR-fluorescent tracers that can detect enzymatic functions with great precision are commercially available. There are tracers for various immune-related targets like Matrix Metalloproteinase (MMP) recognition, angiotensin probes, and neutrophil sensing elastases (134, 135). Optical 3D imaging can be performed using the same imaging probes, which are also used in cell experiments allowing convenient molecular imaging from cells- to the tissue- and organ-imaging without extra labeling steps. While optical tracers per se are relatively small molecules - below 1000 Daltons - and can be coupled with premade linkers, they are easy to use. There are optical tracers for RNA, DNA, proteins, lipids, and carbohydrates. Although contrast agents for fluorescence imaging are optimized for maximal quantum yield some are also applicable in PAI like ICG. However, better molecular tracers are needed for useful immunological 3-D imaging. Fluorescence imaging requires always contrast agents, whereas inflammation imaging with PAI can be used to quantify the increased tissue oxygenation (136), vascularization (137), or fibrosis (138) typical for inflammation. Besides, non-targeted contrast agents like ICG, IRDye, or melanin, or targeted contrast agents like liposomes (139), microbubbles (137), or gold nanoparticles (140, 141) may enhance or specify the photoacoustic signal. Nanoparticle imaging has been shown to offer promising results in immune cell and disease imaging studies (139, 142, 143). The excellent and encouraging results from small animal imaging should be converted to clinical applications in the future. Surgery can benefit from optical imaging with tracers and methods developed for small animal imaging. Surgeons aided by using optical cameras with fluorescent filters in surgical robots and operational microscopies with fluorescent filters allow the better gathering of the visual information on site. Optical imaging could also be used together with endoscopic imaging in gastrointestinal studies to improve IBD treatments significantly.

#### Applications

The human Gastrointestinal (GI) tract microbiota has been a subject of intense research throughout the 3rd Millennium. In recent years, the importance of gut microbe diversity for human health has become evident (144). Robust bacterial clusters, the enterotypes, have been described (145). They are stable bacterial communities composed of a limited number of species. Additional information about bacterial colonizing behavior and metabolism is needed to understand better the relevance of specific strains to human health and diseases like IBD. Fluorescence imaging offers a practical method to understand dynamic interactions between microbe species and microbe-host cells in the gastrointestinal tract. Optical *in vivo* imaging of either bioluminescent or fluorescent bacteria is the basis for non-invasive intestinal colonization detection. The intestine anatomy does not make the GI tract imaging simple, the irregular shape, and most importantly, the deeply embedded organ cause difficulties in 3 D- fluorescence imaging and raise special requirements for the fluorescent markers used. Transcriptional reporters have widely been used in bacterial imaging since Green Fluorescent Protein (GFP)- technology was developed (146). GFP-based imaging has proceeded *in vivo* in the mouse intestine, but the sensitivity does not meet the need to observe bacteria in the physiologically needed range (147). Bioluminescence imaging with luciferases has advantages in sensitivity compared to GFP. Notably, the lux operons are suitable for *in vivo* imaging because there is no need for added substrate, and they have been used in whole-animal imaging in the intestine (148). The background fluorescence from tissues seen in GFP-labeled bacteria can be avoided if Red Fluorescent Proteins (RFP) are used. The dual-color 3D imaging of different bacteria utilizing infrared fluorescent proteins has been presented (149), and several suitable RFPs are available at the moment. Their usage in bacterial imaging has been recently studied by Barbier and Damron (150). They compared the expression, toxicity, photo stability, spectral overlapping, and sensitivity of various fluorescent proteins in *E. coli*. The proteins likeKatushka, mKeima, and E2-crimson (151–153) with red fluorescence are the most promising candidates for the deep tissue *in vivo* applications based on their fluorescence characteristics. The protein toxicity was not a big issue, but instead, spontaneous loss of plasmid in the absence of antibiotics is evident and needs to be considered in study setups. Genetic labels are limited to the bacteria for which cloning tools are available; thus, universal fluorescent labels will offer a powerful tool for proper bacterial imaging. Universal, chemical, fluorescent stains will overcome the question of fluorescence range, while they can be used in higher wavelengths from 640-800 nm, which the fluorescent proteins will not reach. The chemical stains can be based on different chemical interactions. Recently hydrophobic membrane stains have been utilized to label *E. coli* (154). Also, electrostatic interactions can be adapted to label bacteria *in vivo* conditions (155–157). A combination of universal membrane-stain and near far-red fluorescent protein Katushka has also been used successfully with *E. coli* strains. If dual staining is used, the strains can be distinguished from each other, and their mobility can be followed (154). A similar setup could be used in the future to study the interactions of specific bacteria in the colon.

The clinical need is to support the disease diagnostics and evaluate the severity of bacterial inflammation. The most straightforward form of imaging bacteria in the clinical application is to use their endogenous fluorescence by exciting the bacteria with low-intensity violet light (405 nm) (158, 159). In several pre-clinical and clinical bacterial imaging studies, dual radioactive and fluorescent imaging has proceeded mainly using endoscopic set up (160). In these studies, the fluorescent staining was primarily done using bacterial targeting molecules like antibiotics or antibiotic peptides (161, 162), enzyme activated tracers (163, 164), or bacterial lectins (165). In clinical applications, the fluorescent markers cause extra inconvenience, while most of the dyes are not clinically approved. However, few multimodal pre-clinical studies having fluorescent markers as a second marker have been conducted (166, 167). Added fluorescent markers can be used to trace the bacteria from histological samples, differentiate the bacteria type, and evaluate the area of infection and thus aim in the future to image-guided surgery. Though the presented studies are still difficult to implement in clinics, the data collected from multimodal and more theoretical fluorescence studies will, in any case, offer new applications in bacterial diagnostics and treatments. In pre-clinical imaging, PAI has similarly been applied for the detection of different inflammatory diseases such as IBD (168–171), arthritis (172–174), and vascular inflammation (140). With the introduction of the first clinically approved photoacoustic system, the first studies now show the potential for human patient imaging (175).

### Two-Photon Microscopy for Sectioning-Free Virtual Haematoxylin and Eosin (H&E) Imaging

#### Background

In the routine pathology workflow, single-cell layer thick sections of tissue samples required for diagnosis are created by paraffin sectioning. The method is quite a labor and time-intensive process, requiring the sample to be fixed in paraffin for about one day. It is then drained in an automatic machine, usually overnight, which means that water in the tissue is first replaced with alcohol, then with an organic solvent such as xylene, and finally with paraffin. The tissue is then poured into the paraffin and, after cooling, cut into slices of about 5µm thickness using a microtome. These are then placed on a microscope slide. With the help of alcohol, the paraffin is washed out again and usually stained with H&E. The only current established alternative is frozen sectioning, where the sample is embedded in a medium, then flash-frozen and cut into thin slices. The reachable thickness strongly depends on the tissue but is usually a couple of µm thicker than paraffin sections. Artifacts from the freezing process or cutting are a common issue. Although frozen sectioning delivers faster results, the diagnostic quality of the sections is significantly lower than with paraffin sectioning. To establish a faster, less labor-intensive, yet high-quality alternative to thin sections, various optical imaging techniques for the creation of virtual sections have been tested in the research community and some of them were also commercialized (176–181). The tissue sample does not have to be cut, but different optical effects are used to achieve optical sectioning. In most cases, only staining is necessary as sample preparation, which results in a drastic saving of work and time. In a two-photon microscope (TPM) (182) it is exploited that fluorescence can be excited not only by one photon, which can happen anywhere in the light beam, but also by several photons of a lower wavelength that combined have enough energy to excite the fluorophore. Since these photons must be at the same place simultaneously, there is only a sufficient probability for this effect in the focus of the microscope, i.e. in a small spot. The focus can now be moved over the sample to make the dyes fluoresce point by point and create a virtual slice plane.

TPM is a standard tool in neurobiology to observe the activities of nerve cells (183). However, the setups used here usually fill an entire air-conditioned and darkened room. In addition, the titanium-sapphire (Ti : Sa) crystal lasers used are relatively maintenance-intensive, and the existing free beam paths must often be readjusted. A water cooling system is also necessary, which requires regular maintenance. There is one solution, where such a system has been engineered to be used in the clinic (184). We in our group have found that lasers with longer pulse durations in the range of a few 10 ps to a few nanoseconds (SubNs) can also be used for TPM in contrast to the usual ~200fs pulse duration (185). The same images can be obtained at constant average power if the laser’s duty cycle is kept constant, i.e. longer pulses are used, and their repetition rate is reduced by a corresponding factor. The use of longer pulses has the decisive advantage that dispersion in glass fibers no longer plays a major role, and the pulses in these fibers no longer diverge, which would reduce their peak power and thus also the fluorescence signal. For this reason, the laser and the complete beam delivery system up to the microscope optics can now be constructed from glass fibers and corresponding components, which are also used in telecommunications technology. This not only makes the complete setup much more reliable but also less sensitive to temperature fluctuations and vibrations. This enables us to build the entire setup into a mobile rack that can be used anywhere and is also maintenance-free.

#### Applications

**Figure 6:**
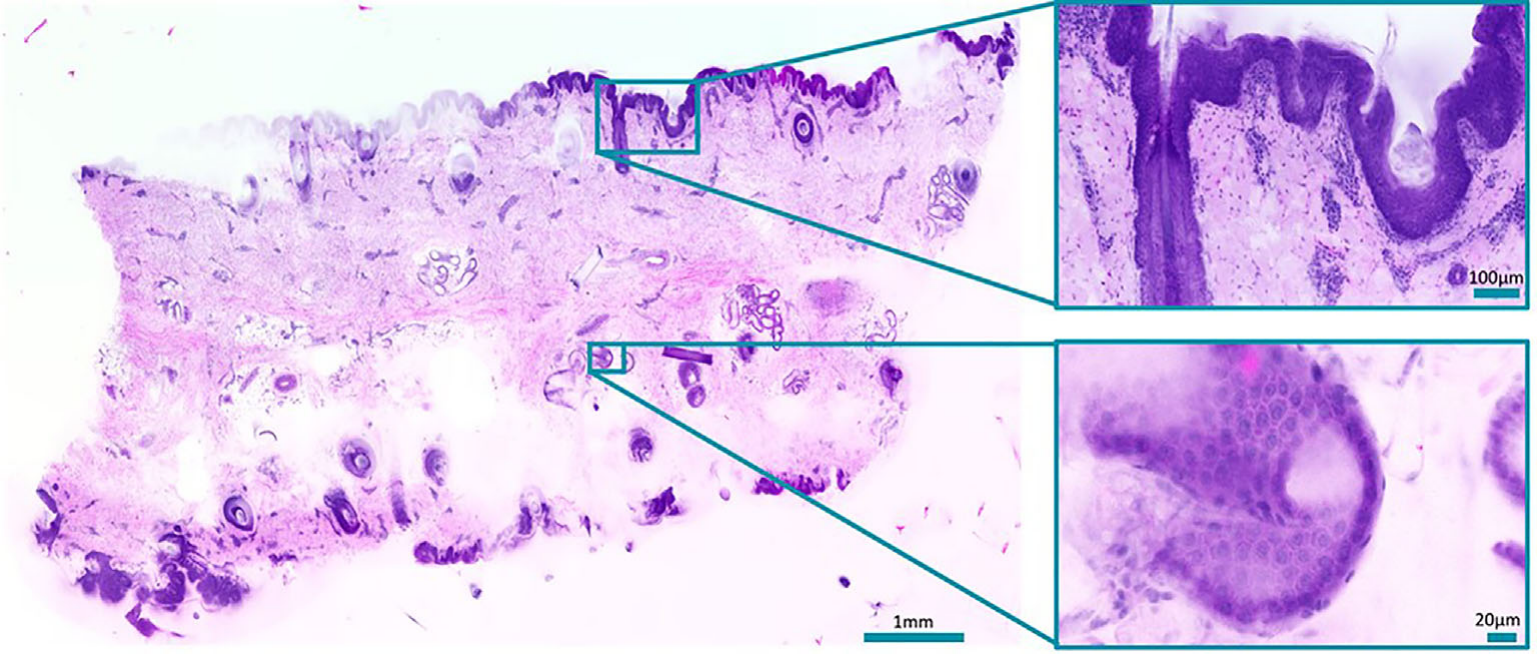
Slide-free image of a bulk porcine skin sample stained with acridine orange and sulforhodamine 101. Zoom-ins show a hair follicle (top) and a sweat duct (bottom). Total acquisition took 13 minutes plus. 10 minutes processing time.

We use TPM of bulk tissue samples to create images that resemble standard H&E-stained slides without any sectioning and to evaluate whether it is a viable alternative. Before imaging, the bulk tissue samples are quick-stained (2-10 min) with acridine orange (nuclei stain) and sulforhodamine 101 (counterstain) to achieve an H&E compatible staining. Our home-built two-photon microscope images the unsectioned tissue samples at high three-dimensional resolution. A plane within the sample is scanned and the fluorescence from the focus is collected by two separate spectral channels to separate nuclei- and counterstain. A digital H&E-equivalent image ready for histological assessment is created from the acquired data. A porcine skin sample was successfully imaged without sectioning using our TPM microscope as seen in **Figure 6**. Compared to the preparation of H&E-stained paraffin sections of the same sample for bright-field microscopy, this took considerably less time and work. Similar image quality and features could be observed compared to paraffin sections. Other types of tissue and more samples are planned to be investigated. Moreover, we intend to further increase the speed of the TPM microscope from currently ~25 minutes/cm² up to 1-2 minutes/cm² with four times more sensitive detectors and by improving the performance of our acquisition and processing software. Also, haematoxylin and eosin (H&E) as stains will be tested to achieve a more realistic image impression. We believe that the pathology workflow can be simplified with virtual H&E imaging with TPM as an alternative to frozen- and paraffin sectioning in the future. The remaining challenges are faster imaging and data processing. It could also provide improved diagnostic accuracy by the potential combination with other imaging modalities (e.g. TPM fluorescence-lifetime-imaging) and the creation of 3D images. Further investigations will include the comparability to standard H&E staining and whether fluorescent immunostains could be used as well.

### Novel Endoscopic Imaging Approaches in Inflammatory Bowel Disease

#### Background

Ulcerative colitis and Crohn`s disease comprise chronic inflammatory bowel diseases that cause severe damage of the integrity of the luminal gastrointestinal tract. The gold standard for the diagnosis of IBD is a combination of clinical presentation, endoscopy, and histology (186). Apart from that, endoscopy in IBD plays a major role in predicting disease severity, extent, and prognosis as mucosal healing was defined as a major therapeutic goal (187). High definition white light endoscopy (HD-WLE) is an important tool in the evaluation of IBD using various endoscopic classification score, i.e. in UC the Ulcerative Colitis Endoscopic Index of Severity (UCEIS) (188) and the endoscopic Mayo score are the ones mainly applied in clinical routine (189). These scores focus on endoscopic findings, such as ulceration, friability of the surface, spontaneous bleeding, and mucosal edema. Since these scores are always limited to the mucosal surface, they exhibit a significant interobserver variability with sensitivities, specificities, and accuracies of 70.8– 95.3%, 67.0–100%, and 32.4–100%, respectively (190) in comparison to histological inflammation as reference (189). Though studies could demonstrate that mucosal healing, assessed after 14 weeks of treatment, correlated with long-term remission in both IBD entities, no commonly accepted definition of mucosal healing has been established so far. Endoscopic findings poorly correlate with histological activity and are not suitable to predict relapse in more individualized therapeutic strategies (190). Therefore, various modern imaging modalities have been explored that enhance detailed mucosa assessments in real-time, including virtual chromoendoscopy techniques, i.e. Narrow-Band Imaging (NBI), Confocal Laser Endomicroscopy (CLE), and Optical Coherence Tomography (OCT).

#### Digital Chromoendoscopy, Narrow-Band-Imaging

Narrow-band-imaging (NBI) utilizes optical filters to illuminate the tissue with defined wavelengths (415 and 540 nm) that are absorbed by hemoglobin but have different penetration depths. NBI, therefore, allows detailed examination of mucosal vascular and surface patterns. In assessing inflammation in IBD and predicting therapy response, divergent data have been published so far. In a prospective study by Kudo et al. 30 UC patients were longitudinally examined, showing good criteria of the Rachmilewitz score and histological markers of inflammation as well as subsequent relapse (191). In contrast, a more recent study in 64 UC patients could not predict relapse within one year of therapy (192). This discrepancy may be explained by the different scoring systems used (Rachmilewitz *vs*. Nishio score) with limitations to superficial criteria.

#### Endoscopic Ultrasound (EUS)

Recent data, in part unpublished, of our group evaluated the role of EUS for the differentiation of CD and UC compared to healthy controls. Combining the EUS criteria total wall thickness (TWT), mucosal/submucosal thickness, and the presence of paracolonic lymph nodes, we could differentiate between active CD and UC with 92.3% sensitivity (193). Furthermore, TWT of the recto-sigmoid colon strongly correlated to histological disease activity prior to initiation of anti-inflammatory therapy and significantly declined within the first two weeks of anti-TNF treatment preceding the changes of the superficial, endoscopic appearance by several weeks. With a sensitivity and specificity of 0.9 a cut-off value of approximately 8% reduction in TWT was calculated to predict therapy response at this very early time point (194).

#### Confocal Laser Endomicroscopy (CLE)

CLE enables real-time imaging of the mucosal surface with ~1000x magnification and a resolution of ~1 micron. It is based on the tissue fluorescence of the target area activated by probe emitting laser light and collecting the emitted fluorescent light at the same time. Hence, CLE requires the use of the intravenous contrast agent fluorescein (1.0–5.0 mL of 10%)., CLE was performed with either an endoscope-based confocal laser endomicroscopy (Pentax, Fort Wayne, NJ, USA; “eCLE”) or a CLE probe (Cellvizio, Mauna Kea Technologies, Paris, France; “pCLE”) that is negotiated *via* the accessory channel of regular endoscopes. However, eCLE is no longer available, even though the majority of confocal applications were studied using it (195). Studies suggest that CLE of intestinal inflammation in IBD can contribute to individualized therapy guidance and predict response and relapse (196). Furthermore, significant progress in molecular *in vivo* imaging may allow exploration of the pathophysiology of IBD and targeted therapies the therapy (197). In study by Li et al. a good correlation between CLE evaluation of crypt architecture and fluorescein leakage with histological findings in subjects with UC was observed. More than 50% of patients with mucosal healing detected during HD-WLE exhibited acute inflammation on histology, whereas no patients in remission confirmed by CLE demonstrated acute inflammation on histology (198). The same group evaluated whether CLE could be used to predict UC relapse in 43 patients with UC. The relapse rate among subjects with CLE-confirmed active disease was significantly higher compared to those with a non-active disease (P < 0.001) (199). CLE has also been studied to specifically determine gastrointestinal (GI) barrier function in patients with IBD (200). Physiologically, intestinal epithelial cells shed from the epithelial layer, whereas new cells migrate from the basal layers in crypts. This gap created by cell shedding can be visualized by CLE and serves as a marker of increased permeability in IBD patients resulting in fluorescein leakage into the lumen (201). Kiesslich et al. observed a significant barrier dysfunction in 47 patients with UC and 11 patients with CD and showed a correlation between intestinal barrier dysfunction and increased risk of relapse (201). Similar results were obtained by Buda et al., who demonstrated that a composite score (Buda score) combining colonic fluorescein leakage with crypt diameter predicts disease flare within one year of follow-up (202).

Our own data suggest CLE-based real-time visualization of blood flow, vascular pattern, and mucosal changes allows an exact quantification of the level of inflammation in IBD. These criteria proved to be reliable to predict early therapy response in patients undergoing anti-integrin therapies (Vedolizumab, VDO) already after two weeks of treatment (203).

### Imaging Cutaneous Inflammation by Optical Coherence Tomography (OCT)

#### Background

Optical biopsy is the concept to replace physical tissue sampling by optically investigating tissue *in vivo* to gain information on pathological changes. One promising approach is optical coherence tomography (OCT), which is a well-established imaging technique in ophthalmology (204). Analogous to ultrasound, OCT uses the reflection of light waves from different tissue interfaces. It measures the propagation time of light by interferometry instead of direct time-of-flight measurements and achieves a higher resolution than ultrasound. OCT is non-invasive, non-contact, fast, and needs no additional marker or contrast agents. Resolution is limited by the spectral band-width of the light source and NA of the imaging optics. Traditionally, most OCT systems provided a resolution of 5 micrometers or worse, which only resolves tissue layers and larger morphology but not cellular structures (205). Changes in these larger structures due to inflammatory processes can be visualized and quantified in cross-sectional or even volumetric images (206–208). Due to the use of interferometry in the imaging process, OCT also depicts very sensitively local motion. This enables a marker-free angiography which visualizes vessels down to the capillary level (209, 210).

**Figure 7:**
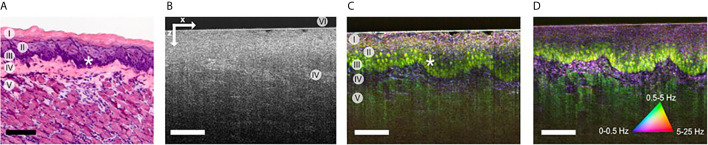
**(A)** HE stained histology of the imaged sample at different location (I) cornified layer, (II) granular & spinous layers, (III) basal layers, (IV) lamina propria, (V) muscle, and (VI) glass plate. **(B)** OCT image of mouse tongue; lamin propira (IV) can be identified by brighter contrast. **(C)** Corresponding dynamic contrast mOCT image with a focus in basal layer (I-V) and even nuclei (*) are visible. **(D)** Dynamic contrast m OCT image with a focus in the lamina propria; the image size is 380x500 μm (zx); scale bar, 100 μm [from ref. (221)].

Very high resolution OCT systems have been investigated in the past (211–215), but only recently their full potential has been demonstrated for cellular imaging (216). At a resolution better than 2 μm tissue structures on cellular and subcellular level become visible (216–218). Besides resolution, imaging contrast is also important. Contrary to fluorescence imaging, OCT lacks a cell-specific contrast. Neither are specific marker available. However, transferring the principle of OCT angiography to higher resolution and longer time scales, a cell and tissue specific contrast was introduced. It was first demonstrated with FF-OCT for en-face images (219, 220) and recently also using scanning OCT for cross-sectional imaging (221). The contrast is based on microscopy intra-cellular motion, which in general caused by structures below the imaging resolution, but is detected by the interferometric imaging process on which OCT is based. Combining microscopic resolution and dynamic motion contrast individual cells and connective tissue are visible with a fluorescent-like contrast (**Figure 7**). Since microscopic motion is the basis of contrast, general tissue motion destroys the contrast. Mechanical stabilization of the tissue is crucial and currently in-vivo imaging has yet been demonstrated. Analyzing cellular morphology and dynamic processes of immune cells may, in the future, enable a marker-free optical biopsy of inflammatory processes by OCT.

In ophthalmology, OCT has become standard for retinal diagnosis and is also clinically used for imaging the anterior segment of the eye. The unique properties of OCT which provides micrometer lateral and axial resolution despite the limited pupil size of the eye, make OCT the only imaging technique, which can visualize and quantify the layered structure of the retina. The retina offers unique optical access to neuronal tissue and the microcirculation and gives opportunity to diagnose and quantify systemic neuronal and vascular diseases. Diagnostic applications include inflammatory diseases like lupus, systemic sclerosis, Behçet disease, spondylitis, and familial Mediterranean fever (208). OCT and OCT angiography are also valuable tool in inflammation diagnosis of the anterior segment (222, 223). The clinical applications of OCT in areas other than ophthalmology are currently found in skin imaging, cardiovascular imaging, and gastroenterology, as only there clinically approved OCT devices are commercially available. Previously, most dermatology studies dealt with the visual presentation of tumor diseases and only few papers evaluated OCT’s potential for inflammatory diseases (206, 224–226) In gastrointestinal diseases the first results from endoscopic optical biopsy were published 20 years ago (227). OCT could be used to identify transmural inflammation and morphological differentiation between UC and CD from patient ex vivo tissue samples (228) and *in vivo*, providing a valuable tool to distinguish CD from UC (229). This is especially relevant since biopsies are insufficient to assess for transmural inflammation. Although these data are encouraging, subsequent confirmation in larger, longitudinal follow-up trials is missing so far.

Definitely the potential of OCT is not yet exhausted in the field of inflammation. Especially, the significant increase in imaging speed and imaging resolution in last year gives new options for imaging inflammatory processes on a cellular level. A response to treatment could be detected early to enable individual adaptation of the treatment strategy through the accurate representation of the inflammatory processes. If molecular and cellular changes are detected at an early stage of disease progression or in the treatment of inflammatory diseases, in that case, it is possible to make and optimize individual treatment decisions. The following section will concentrate on dermatological applications of OCT, which is currently the only field in which commercial OCT devices with cellular resolution are available of clinical diagnosis (230).

#### Applications

The skin is the largest organ of the human body. In the clinical routine, the patient’s skin is firstly examined with the naked eye. Conspicuous skin lesions can be further assessed with the help of dermoscopy, which allows the magnification of the skin surface and the superficial vessels. Skin alterations, including cellular and deep vascular changes, typically require tissue removal for histological examination. Histology is the gold standard diagnostic method. However, invasiveness, expenditure of time, limitation to two-dimensional sectioning, and lack of monitoring dynamic changes make the histological examination evidently improvable. Hence, there is a demand for non-invasive methods that enable real-time, three-dimensional, and *in vivo* imaging of the skin.

OCT has the potential to combine fast bedside imaging with the opportunity to monitor therapeutic effects (224). There are reports on applications of OCT in dermatology mostly for skin tumors (231), but rarely for inflammatory skin diseases (232) and evaluation of treatment effects (233). Vascular alterations can be detected using OCT angiography, also known as dynamic OCT (234). Since 1997, reports related to OCT in dermatology have increased (235). This implicates the growing importance of OCT for clinical applications. Other well-established, *in vivo* imaging modalities that could be compared to OCT are ultrasound (236), confocal microscopy (237), multiphoton tomography (238), and magnetic resonance imaging (239). The highest comparability with regard to resolution and penetration depth is with OCT and high-frequency ultrasound. Both methods provide time- and cost-effectiveness. Also, OCT could be widely available in the future. The resolution typically decreases with higher penetration depth. Ultrasound imaging exhibits high tissue penetration visualizing fat and muscle, but the resolution is lower compared to OCT (236). Inflammation changes the tissue composition leading to higher water content and lower collagen content. Therefore, it has been shown for OCT that the signal penetration could even be increased due to lower scattering. In contrast, inflammatory processes and edema lead to a signal decrease for ultrasound (225).

**Figure 8:**
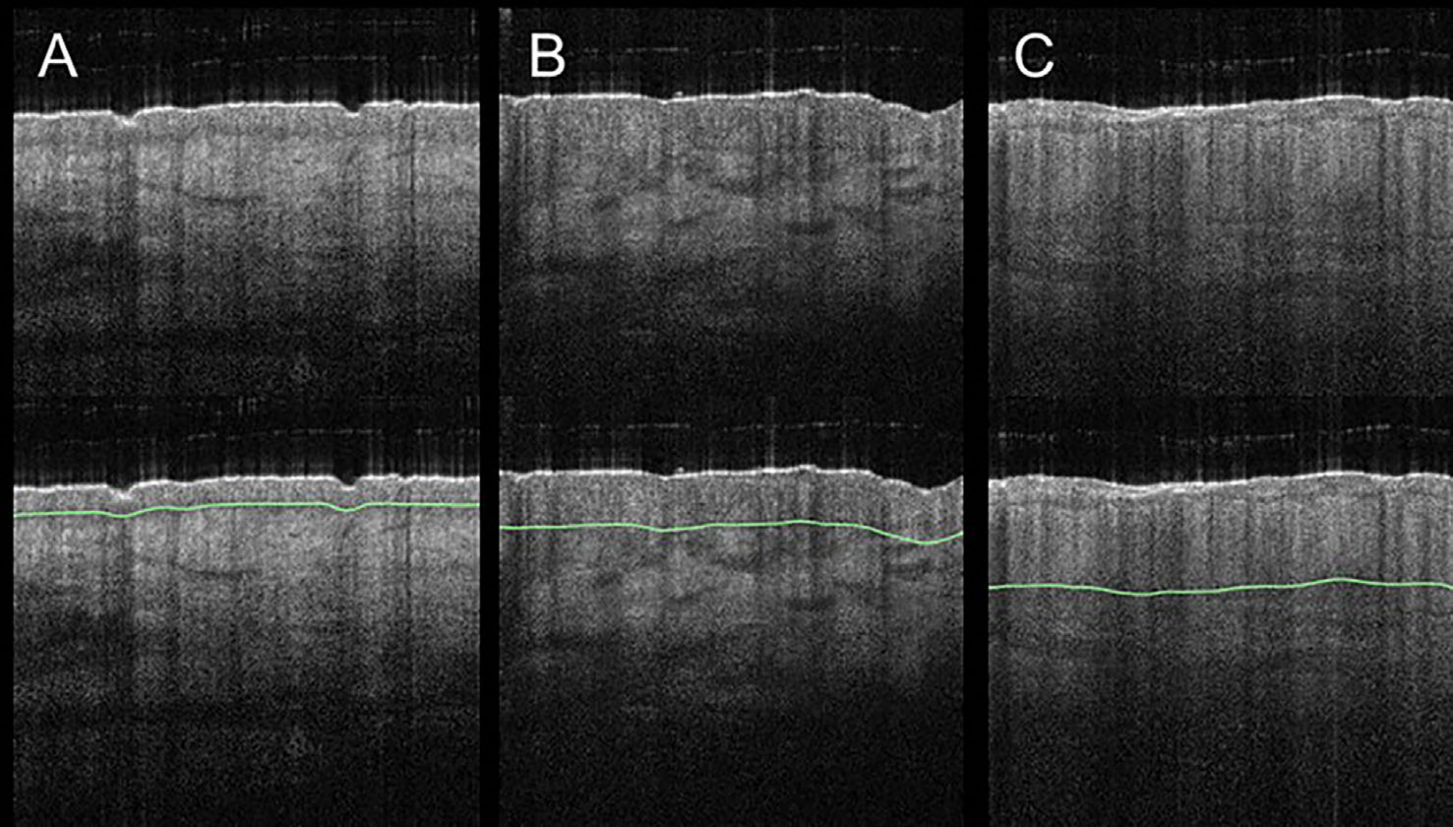
Acute and chronic inflammatory skin diseases can lead to an increase of the epidermal layer. Compared to healthy skin **(A)**, involved skin in atopic dermatitis **(B)** and in plaque psoriasis **(C)** exhibit a thicker epidermal layer. Changes of epidermal thickness (green line) can be visualized in vertical B-scans and measured by OCT.

**Figure 9:**
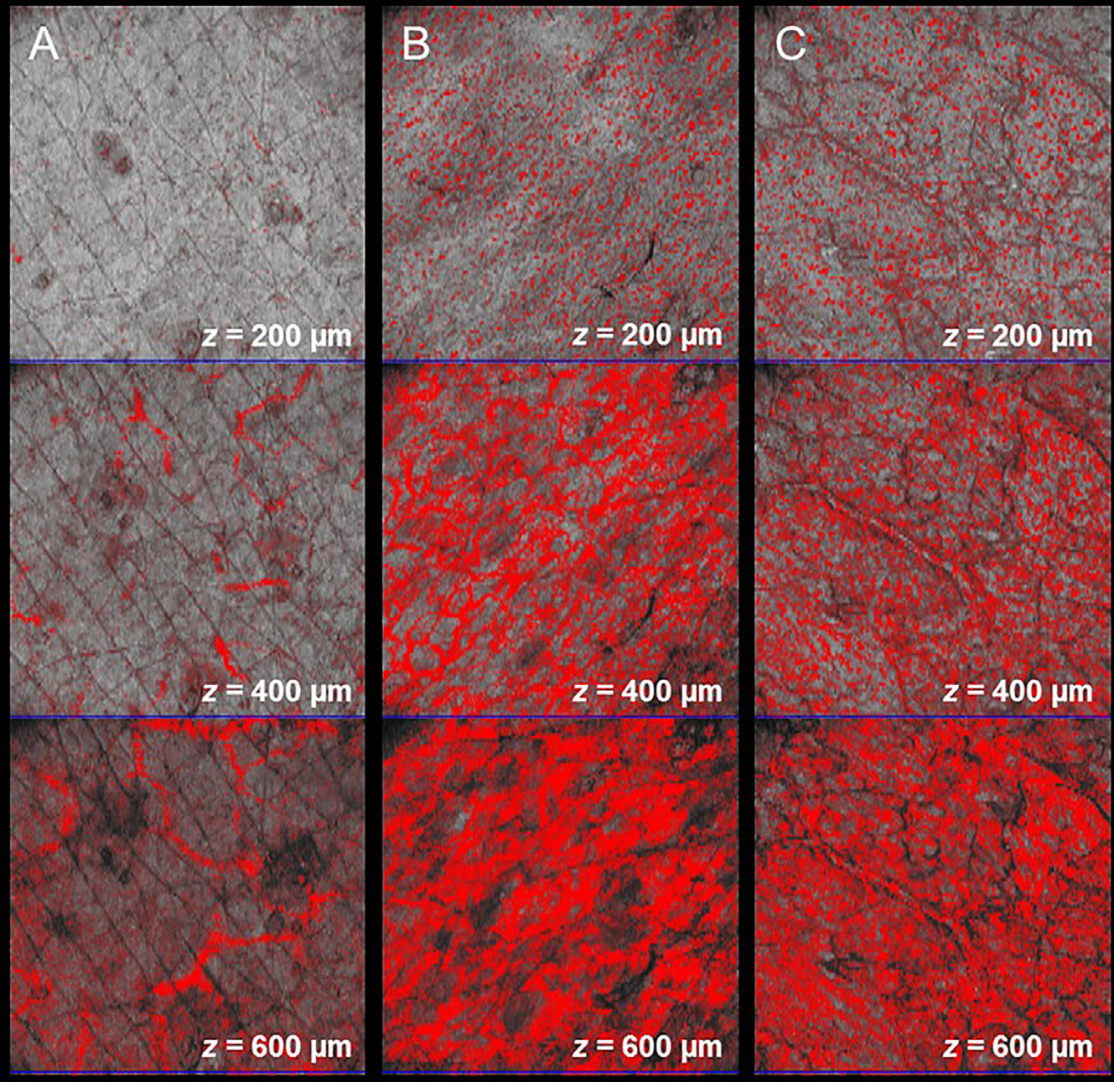
Angiographic OCT allows the visualization of elongated capillary loops in the superficial papillary dermis and the underlying vessel plexus. In comparison to the healthy control **(A)**, changes of vascular pattern, vessel diameter, depth, and density can be observed in lesional skin in atopic dermatitis **(B)** and in plaque psoriasis **(C)**.

Skin diseases that result in structural and vascular changes can be determined and quantified in OCT. For example, atopic dermatitis and plaque psoriasis are common inflammatory diseases that lead to a higher first intensity peak in the A-scan due to higher reflectivity (225). Further, the second intensity peak is correlated with alterations of the dermal-epidermal junction. The efficacy of potent biologic treatments could be assessed using OCT. Imaging parameters could be skin structure, epidermal thickness (**Figure 8**), entrance peak, dermal reflectivity, attenuation coefficient, plexus depth, vessel diameter, density, and tortuosity (**Figure 9**) (224, 240).

OCT can be applied for monitoring psoriasis treatment. In the near future, skin assessment with the use of OCT could become an inherent part of the clinical routine (233). Finally, the development of an OCT-based inflammation score system with regard to skin structure and perfusion could allow more tailored treatment opportunities. The goal of precision medicine is the gain of more predictive information from OCT data on early treatment response or treatment failure of current biologic treatments.

## Conclusions

Imaging inflammation is a key component to understand and treat the various manifestations of the disease. Virtually all modern imaging technologies have identified inflammation as a goal and are developed methods to image it. The overview in this article shows that different modalities are needed to tackle different aspects of the disease. Impressive progress has been made and will continue, bringing precision medicine to life.

In tumor imaging and analytics, several new methods have been tested in clinics. These methods could be brought into use quite easily in many cases of inflammation disease as well. Like, cancer imaging techniques might solve problems that appear in inflammation imaging today. Overall the problem of optical imaging in medicine in humans is the shallow penetration. The solutions might include guiding the light deep into the body endoscopes or optical fibers and develop better imaging tracers. Also, photoacoustic might be a valuable method in future imaging. Optical 3D imaging offers the best platform for extending molecular imaging from cells to the tissue and organ level. OCT can be used to see micro-structures below the surface, scan larger areas, quantify inflammation by geometry measurements, and visualize blood flow. Thus OCT could be used to image the structural tissue changes in IBD. PET and SPECT, both being available for humans and rodents, can play a key role in translating the knowledge gained in preclinical research into the clinics. These two imaging techniques are being already regularly used in macrophage detection and, in the case of FDG-PET, also for energy consumption in inflammations; however, further opportunities are expected if more specific molecular targets with adequate radiolabeling are developed. All of the imaging modalities would benefit from new, better, and more specific tracers. New solutions in MRI would serve the possibility to track inflammation based on metabolic activity differences between normal and inflamed tissues.

A combination of different imaging modalities would simultaneously offer information about the structure, success of targeting, or metabolic activity from the tissue of interest. In fact, multimodality has already reached the clinical and preclinical environments, e.g. through PET/CT and PET/MRI. Further benefits could be achieved by developing bi- or even multimodal imaging agents. Although bimodal probes have been proposed for PET/MR as well as for PET and optical imaging, they still remain less specific than the probes designed for each single modality. In addition to diagnostic purposes, imaging could be an extra tool in medical operations like in surgery. There are indeed several concepts for intraoperative optical imaging like optical fluorescent operational microscopes, fluorescent cameras for robotic surgery, OCT integrated surgical microscopes, as well as methods based on radioactive signal detection. Among the latter, gamma, beta minus, and beta plus probes, mini gamma cameras, intraoperative PET detectors and freehand SPECT have proposed. We have gathered the current clinical and relevant assortment of potential preclinical tracers of different imaging modalities as a table (**Table 1**). Some of these tracers, especially nano-probes, could be easily converted to multimodal tracers. While some of these methods have already reached the clinical arena, some others are still under investigation. Here additional information collected by different imaging modalities would help to solve multiple current unmet needs in inflammation like in robotic real-time surgery operations.

**Table 1:** Current clinical and relevant assortment of potential preclinical tracers of different imaging modalities have been gathered to the table.

Technique	Disease/organ	Marker	Preclinical/Clinical	References number
**ASL-MRI**	stroke / brain	cerebral blood flow; arterial transit time	Clinical	(241–243)
	stroke / pediatric brain	cerebral blood flow	Clinical	(244–246)
	tumors / brain	cerebral blood flow	Clinical	(247, 248)
**DCE-MRI**	multiplesclerosis (MS)/Brain	blood-brain barrier permeability; volume transfer constant; extracellular space volume fraction	Clinical	(249)
	stroke / brain	blood-brain barrier permeability; contrast-agent leakage rate; volume transfer constant	Clinical	(250)
**DCE-MRI**	multiplesclerosis (MS)/Brain	BBB permeability	Clinical	(19)
	prostatic hyperplasia / prostate	perfusion fraction; extraction fraction; mean transit time; extravascular-extracellular volume	Clinical	(18)
**DSC-MRI**	tumors / brain	cerebral blood volume	clinical	(14, 15)
	stroke / brain	cerebral blood flow; cerebral blood volume; mean transit time	clinical	(243)
**MRI**	Vasculitis/central nervous system/brain	arterial wall thickening, vessel wall enhancement on post-gadolinium black-blood MRI	Clinical	(33–37)
	Intracranial aneurysms/brain	aneurysm wall enhancement on post-gadolinium black-blood MRI	clinical	(42–46, 53–56)
	IBD / small bowel and colon	bowel wall thickening, restricted diffusion, edema, increased contrast media uptake, strictures, ulcerations, reduced motility, mesenterial reaction	clinical	(59–64, 66–69)
**metabolic hyperpolarized MRI**	arthritis	anaerobic glycolysis; lactate; lactate dehydrogenase	Preclinical/Clinical	(79)
	liver	anaerobic glycolysis; lactate; lactate dehydrogenase	preclinical	(80)
	myocardial infarction	anaerobic glycolysis; lactate; lactate dehydrogenase	Preclinical/Clinical	(81)
	muscullo skeletal	anaerobic glycolysis; lactate; lactate dehydrogenase	preclinical	(82)
	general inflammation	pH; bicarbonate/CO2, zymonic acid; pyruvate dehydrogenase	preclinical	(251)
				
**gaseous hyperpolarized MRI**	lung, brain	ventilation, dissolved-phase imaging; ^129^Xe	clinical	(83)
	head, neck, lungs	FLASH) MRI; ^3^He, proton (H_2_O, CH_2_-group)	pre-clinical/clinical	(84)
	COPD/lung, asthma, cystic fibrosis	ventilation, diffusion and dissoved-phase imaging; ^129^Xe, ^3^He; Oxygen concetration, lung capacity.	clinical	(85)
	lung, kidney, brain/ COPD	Ventilation, dissolved-phase imaging; ^129^Xe, ^3^He; barrier uptake, red blood cell transfer, ventilation defect percentage.	clinical	(86)
	lung/ emphysema	diffusion imaging; ^3^He; apparent diffusion coefficient.	clinical	(87)
	lung/COPD, Idiopathic pulmonary fibrosis, left heart failure, pulmonary arterial hypertension	Ventilation and gas transfer maps ; ^129^He; ventilation defects, Red blood cell- transfer.	clinical	(88)
	lung/ COPD	Transfer Contrast MRI; ^129^Xe; apparent diffusion coefficient.	clinical	(89)
	lung/ COPD, asthma	3D dissolved-phase imaging; ^129^Xe; red blood cell to tissue–plasma ratio.	clinical	(90)
	lung/ idiopathic pulmonary fibrosis	Spectroscopic imaging; ^129^Xe; regional gas exchange.	clinical	(91)
	lung/ COPD, asthma	2D and 3D ventilation imaging, ^129^Xe; dissolved-phase to gas-phase xenon ratio.	clinical	(92)
				** **
**SPECT/PET**				** **
	IBD /and many other inflammations	18F-FDG	clinical	(94, 102–117)
	IBD	Leukocytes99mTc-HMPAO-leukocytes	clinical	(252)
	IBD	CXCL899mTc-CXCL8	clinical	** **
	IBD	β764Cu-FIB504.64-Fab	Preclinical	** **
	IBD	α4β764Cu-DATK32	Preclinical	** **
	IBD	β764Cu-FIB504.64-Fab	Preclinical	** **
	IBD	β764Cu-FIB504.64-F(ab′)2 (fragments)	Preclinical	** **
	IBD	CD489Zr-GK1.5 cys-diabody	Preclinical	** **
	IBD	TNF-α99mTc-InfliximabRatsTNBS	Preclinical	** **
	IBD	IgG111In-IgGRabbitsTNBS	Preclinical	** **
	IBD	Leukocytes111In-WBC	Preclinical	** **
	IBD	Liposomes111In-liposomes	Preclinical	** **
	IBD	IL-899mTc-HYNIC-IL-8RabbitsTNBS	Preclinical	** **
	IBD	Granulocytes99mTc-HMPAO-Granulocytes	Preclinical	** **
	IBD and many inflammations	imaging inflammatory cells	Preclinical	(253)
	IBD and many inflammations	11C-PK11195,	Preclinical	** **
	IBD and many inflammations	18F-FEDAA1106,	Preclinical	** **
	IBD and many inflammations	18F-FEMPA,	Preclinical	** **
	IBD and many inflammations	18F-GE-180,	Preclinical	** **
	IBD and many inflammations	68Ga-DOTATATE,	Preclinical	** **
	IBD and many inflammations	64Cu-DOTATATE,	Preclinical	** **
	IBD and many inflammations	68Ga-DOTANOC,	Preclinical	** **
	IBD and many inflammations	18F-FDR-NOC,	Preclinical	** **
	IBD and many inflammations	68Ga-DOTATOC,	Preclinical	** **
	IBD and many inflammations	64Cu-DOTA-DAPTA-comb nanoparticles, 64Cu-DOTA-ECL1i,	Preclinical	** **
	IBD and many inflammations	64Cu-DOTA-vMIP-II,	Preclinical	** **
	IBD and many inflammations	64Cu-vMIP-II-comb nanoparticles,	Preclinical	** **
	IBD and many inflammations	18F-FOL Folate receptor βMacrophages,	Preclinical	** **
	IBD and many inflammations	68Ga-NOTA-MSA,	Preclinical	** **
	IBD and many inflammations	18F-FDM,	Preclinical	** **
	IBD and many inflammations	64Cu-MMR and 68Ga-MMR nanobodies,	Preclinical	** **
	IBD and many inflammations	18F-fluorothymidine,	Preclinical	** **
	IBD and many inflammations	18F-fluoromethylcholine,	Preclinical	** **
	IBD and many inflammations	11C-choline,	Preclinical	** **
	IBD and many inflammations	68Ga-Fucoidan,	Preclinical	** **
	IBD and many inflammations	64Cu-DOTA-anti-P-selectin antibodies,	Preclinical	** **
	IBD and many inflammations	18F-4V,	Preclinical	** **
	IBD and many inflammations	64Cu-VCAM nanobody,	Preclinical	** **
	IBD and many inflammations	18F-HX4,	Preclinical	** **
	IBD and many inflammations	18F-FMISO,	Preclinical	** **
	IBD and many inflammations	62Cu-ATSM,	Preclinical	** **
	IBD and many inflammations	18F-fluciclatide,	Preclinical	** **
	IBD and many inflammations	18F-Galacto-RGD,	Preclinical	** **
	IBD and many inflammations	18F-Flotegatide,	Preclinical	** **
	IBD and many inflammations	64Cu-DOTA-C-ANF,	Preclinical	** **
	IBD and many inflammations	DOTA-CANF-comb nanoprobe,	Preclinical	** **
	IBD and many inflammations	18F-florbetaben,	Preclinical	** **
	IBD and many inflammations	18F-flutemetamol,	Preclinical	** **
	IBD and many inflammations	68Ga-DOTATATE,	Preclinical	** **
	IBD and many inflammations	18F-FET-βAG-TOCA	Preclinical	** **
**Optical/PAI**	rheumatoid arthritis	ICG blood flow indicator	Clinical	(131)
		Many tracers examples aMSH, MMP binding tracer, RGD,	Preclinical	(133–143)
		VEGF targeting nanoparticles, ASMase targeting liposome,		** **
		Alendronate targeted nanoparticles		** **
	IBD	Hemoglobin	preclinical	(168)
	CD	Hemoglobin and fibrosispreclinical		(169–171)
	Arthritis	L-selectin/P-selectin-targeting contrast agent	preclinical	(172)
	Arthritis	Hemoglobin	preclinical	(172–175)
	Atherosclerosis	gold nanorods conjugated with MMP_2_ antibody	preclinical	(140)
	Wound	Endogeneous bacterial fluoresence	Preclinical	reviewed in (158)
	Invasive- and biomaterial-associated bacterial infections	conjugated vancomycin to IRDye	Early clinical trial	(161, 167)
	Tuberculosis	fluorogenic substrates for beta-lactamase	preclinical	(164)
	Wound infection	Lectin base fluorescent nanoparticle	preclinical	(165)
	Bacterial infections (implants)	Antimicrobial peptide conjugated to a radioisotope and a fluorescent dye	preclinical	(166)
	Not limited to certain organs or diseases. Can be used to measure morphological changes of tissue.	Fluorecenct stains and/or autofluorecence	Certified medical devices for in vivo skin measurements avaible. Other applications are preclinical.	(184)
**Digital chromoendoscopy**	IBD/colon	Mucosal surface patterns	Clinical	(191, 192)
**Endoscopic ultrasound**	IBD/colon	Total wall thickness, mucosal vascularity	Clinical	(193, 194)
**Confocal laser endomicroscopy**	IBD/colon	Crypt diameter, fluorescein leak, mucosal vascularity	Clnical	(195–203)
				** **
**TPM**	Not limited to certain organs or diseases. Can be used to measure morphological changes of tissue.	Fluorecenct stains and/or autofluorecence	Certified medical devices for in vivo skin measurements avaible. Other applications are preclinical.	(184, 236, 238)
**OCT**	Human eye		Clinical	(204, 205, 208, 210, 222, 223),
	Human coronary artery		Clinical	(205, 216)
	Human oesophagus		Clinical	(205, 207)
	Human small intestine		Clinical	(205, 207)
	Human colon		Preclinical	(205, 207, 214, 228, 229)
	Human biliary and pancreatic ducts		Clinical	(205, 207)
	Human lung		Preclinical	(205, 217, 218)
	Skin		Clinical	(205, 212, 224–226, 230–235, 239, 240)
**confocal microscopy**	skin		Clinical	(233, 237)

The newly-developed research techniques are tested in clinical and preclinical studies to improve diagnosis and identify the individual patient response to treatment at an early stage in precision medicine. The close cooperation of engineering expertise with clinical applications leads to further developing state-of-the-art imaging methods in inflammation medicine. Modern optical microscopy already enables microscopically small cell changes to be identified and assessed in real-time. The existing experimental methods can only be transferred into a clinical application with direct benefits for the patient by close dialogue between the different scientific disciplines.

## Author Contributions

RH, GH, OP, TP, and JH wrote the optical imaging part. RH, GH, J-BH, MR, OP, and TP wrote abstract, introduction, and conclusions and did proofreading for the review. TP put the text together and was a contact person for the writers. JEH, LH, RH, GH, and ME made the OCT part. JK, GH, and RH provided the TP part. MR and OP wrote PET/SPECT part. J-BH, MB, NL, PU, AF, MSP, and MA provided the MRI part. ME contributed writing the endoscopic approaches in inflammatory bowel disease. All authors contributed to the article and approved the submitted version.

## Funding

This research was funded by the European Union project within interreg Deutschland-Denmark from European Regional Development Fund (ERDF) (CELLTOM), German Science Foundation (DFG HU 629/6-1), and the German Ministry of Research, Innovation and Science (82DZL001A2), Bundesministerium für Bildung und Forschung (BMBF-Neuro-OCT no. 13GW0227B), State of Schleswig Holstein (Excellence Chair Program), Deutsche Forschungsgemeinschaft (EXC 2167-390884018, HU1006/6 27087.1130), ATTRACT project funded by the EC under Grant Agreement 77722, DAMP Foundation, and funding from (DFG PMI2167), FOR5042, GRK2154, TRR287, and ho 4604/3-1.

## Conflict of Interest

The authors declare that the research was conducted in the absence of any commercial or financial relationships that could be construed as a potential conflict of interest.

## Abbreviations

ASL, Arterial Spin Labelling; BBB, Blood-Brain Barrier; BB, MRI Black-Blood MR imaging; CA, Contrast-Agent; CBF, Cerebral Blood Flow; CBV, Cerebral Blood Volume; CD, Crohn’s Disease; CLE, Confocal Laser Endomicroscopy; CNS, Central Nervous System; CT, Computed Tomography; DCE-MRI, Dynamic Contrast-Enhanced; DKI, Diffusion Kurtosis Imaging; DSA, Digital Subtraction Angiography; DSC-MRI, Dynamic Susceptibility Contrast; DSS, dextran sodium sulfate; DWI, Diffusion- Imaging; EUS, Endoscopic Ultrasound; FDG, Flurodeoxyglucose; FSE, Fast Spin-Echo; GFP, Green Fluorescent Protein; GI, Gastrointestinal; H&E, haematoxylin and eosin; IBD, Inflammatory Bowel Diseases; ICG, Cardiogreen; MaRIA score, Magnetic Resonance Index of Activity; MI, myocardial infarction; MMP, Matrix Metalloproteinase; MRI, Magnetic Resonance Imaging; MTT, Mean Transit Time; NBI, Narrow-Band Imaging; NIR, Near-Infrared; OCT, Optical Coherence Tomography; PACNS, Primary Angiitis of the CNS; PAI, Photoacoustic Imaging; PALM, Photo‐Activated Localization Microscopy; PCASL, pseudo-continuous ASL; PET, Positron Emission Tomography; RFP, Red Fluorescent Proteins; SNR, Signal to Noise Ratio; SPECT, Single Emission Computer Tomography; SSFP, GE Steady-State Free Precession Gradient Echo; STED, Stimulated Emission Depletion; TB, Total-Body; 3D T1 FSE 3-dimensional, T1-weighted fast spin-echo sequence; TNF, Tumor Necrosis Factor; TPM, Two-Photon Microscope; UC, Ulcerative Colitis; UCEIS, Ulcerative Colitis Endoscopic Index of Severity; US, ultrasound; VDO, Vedolizumab; VWI, Vessel Wall Imaging.
